# Comparison of effect estimates between preprints and peer-reviewed journal articles of COVID-19 trials

**DOI:** 10.1186/s12874-023-02136-8

**Published:** 2024-01-11

**Authors:** Mauricia Davidson, Theodoros Evrenoglou, Carolina Graña, Anna Chaimani, Isabelle Boutron

**Affiliations:** 1 Center for Research in Epidemiology and Statistics (CRESS-U1153), Université Paris Cité and Université Sorbonne Paris Nord, INRAE, Inserm, Hôpital Hôtel-Dieu, 1 Place du Parvis Notre-Dame, Paris, F-75004 France; 2 Centre d’Epidémiologie Clinique, AP-HP, Hôpital Hôtel Dieu, Paris, F-75004 France; 3 Cochrane France, Paris, France

**Keywords:** Preprint, Peer-review, Discrepancy, COVID-19, Randomized controlled trial

## Abstract

**Background:**

Preprints are increasingly used to disseminate research results, providing multiple sources of information for the same study. We assessed the consistency in effect estimates between preprint and subsequent journal article of COVID-19 randomized controlled trials.

**Methods:**

The study utilized data from the COVID-NMA living systematic review of pharmacological treatments for COVID-19 (covid-nma.com) up to July 20, 2022. We identified randomized controlled trials (RCTs) evaluating pharmacological treatments vs. standard of care/placebo for patients with COVID-19 that were originally posted as preprints and subsequently published as journal articles. Trials that did not report the same analysis in both documents were excluded. Data were extracted independently by pairs of researchers with consensus to resolve disagreements. Effect estimates extracted from the first preprint were compared to effect estimates from the journal article.

**Results:**

The search identified 135 RCTs originally posted as a preprint and subsequently published as a journal article. We excluded 26 RCTs that did not meet the eligibility criteria, of which 13 RCTs reported an interim analysis in the preprint and a final analysis in the journal article. Overall, 109 preprint–article RCTs were included in the analysis. The median (interquartile range) delay between preprint and journal article was 121 (73–187) days, the median sample size was 150 (71–464) participants, 76% of RCTs had been prospectively registered, 60% received industry or mixed funding, 72% were multicentric trials. The overall risk of bias was rated as ‘some concern’ for 80% of RCTs. We found that 81 preprint–article pairs of RCTs were consistent for all outcomes reported. There were nine RCTs with at least one outcome with a discrepancy in the number of participants with outcome events or the number of participants analyzed, which yielded a minor change in the estimate of the effect. Furthermore, six RCTs had at least one outcome missing in the journal article and 14 RCTs had at least one outcome added in the journal article compared to the preprint. There was a change in the direction of effect in one RCT. No changes in statistical significance or conclusions were found.

**Conclusions:**

Effect estimates were generally consistent between COVID-19 preprints and subsequent journal articles. The main results and interpretation did not change in any trial. Nevertheless, some outcomes were added and deleted in some journal articles.

**Supplementary Information:**

The online version contains supplementary material available at 10.1186/s12874-023-02136-8.

## Background

The scientific community has witnessed a significant shift in the way research findings are disseminated due to the COVID-19 pandemic and the subsequent rise of preprints [1, 2]. Preprints are early versions of scientific research papers that are made publicly available before they have undergone formal peer review and publication. By circumventing the lengthy peer review process, preprints allow for rapid communication on new evidence to inform public health responses. This is particularly crucial during pandemics. Notably, results of the world’s largest COVID-19 platform trial, RECOVERY [3], were first reported as preprints, enabling swift, real-time evaluation of the interventions and potential harms. While discussing the benefits of preprints in patient care, lead RECOVERY author, Peter Horby, emphasized that peer review delays could have life-threatening consequences [4].

Without formal peer review and rigorous quality control, preprints can amplify misleading information stemming from biases, methodological limitations, incomplete analyses, and even fraud [5]. Preprint use has been scrutinized both from a public understanding perspective and in regards to scientific principles. Firstly, there is a concerning lack of understanding of preprint data among the general public. For example, widespread media attention given to two small, biased preprints that erroneously claimed smoking to be protective against COVID-19 impacted public health as it resulted in a surge in nicotine purchases and smoking uptake in certain countries [6].

Secondly, it is reasonable to expect some discrepancies between the content of various documents and sources for the same randomized controlled trial (RCT), particularly between the preprint and the subsequent journal article, as peer review often impacts the content of a manuscript before it is published. A meta-research study of 139 studies reported in preprint and subsequent journal article or in different versions of the preprint found a change in the abstract’s conclusion in 24% of studies [7]. In contrast, a study of 78 preprint–article pairs of RCTs showed consistency in terms of the completeness of reporting [8]. Another analysis of 67 interventional and observational studies found that preprints and their subsequent journal articles were similar in terms of reporting and spin (i.e., distorted interpretation of results) [9]. Similarly, a study of 74 preprint–article pairs of RCTs showed few important differences in treatment effect estimates between the two documents [10].

To further explore the consistency between various documents reporting the results of trials, we assessed the consistency in effect estimates between preprints and subsequent journal articles of COVID-19 RCTs included in a large living systematic review of COVID-19 pharmacological treatments.

## Methods

The protocol is available on Open Science Framework (https://osf.io/hfrp4/?view_only=b06282a8429e4ae1af458f4e372576f7). Here, we report the results of objective one - to assess the consistency in the estimates of treatment effects between preprints and the subsequently published articles. We expanded our sample size by including RCTs assessing all pharmacological treatments instead of limiting our analysis to specific treatment types as planned in the protocol. Additionally, we updated the final search to July 20, 2022.

### Data source and search

Our study used the data and methods of the COVID-NMA living systematic review (covid-nma.com) [11] [see Methods S1 in the Additional file]. Briefly, COVID-NMA is a living evidence synthesis and living mapping of RCTs on interventions for the prevention and treatment of COVID-19. The search strategy was modified over time to involve searching only two bibliographic databases: the Epistemonikos L-OVE COVID-19 platform [12] and Cochrane COVID-19 Study Register [13]. The Retraction Watch database [14] was also searched to identify retracted trials and directly remove them from the COVID-NMA review (Additional file Table S1). Screening and data extraction were performed by pairs of researchers, independently and in duplicate, with disagreements resolved by consensus and a third researcher, when necessary.

### Eligibility criteria

We selected eligible RCTs in the COVID-NMA living systematic review that evaluated pharmacological treatments for patients with COVID-19 and that were originally posted as preprints and subsequently published in a peer-reviewed journal. The last search date was July 20, 2022. We considered the following COVID-NMA-defined critical outcomes:

- Clinical improvement at day 28 (D28) defined as a hospital discharge or improvement on the scale used by trialists to evaluate clinical progression and recovery.
- WHO Clinical Progression Score of level 7 or above (i.e., mechanical ventilation +/– additional organ support or death) (D28).
- All-cause mortality (D28).
- Incidence of any adverse events.
- Incidence of serious adverse events.

We excluded RCTs evaluating preventive interventions (e.g., use of personal protective equipment, movement control strategies), vaccines, non-pharmacological treatments, and supportive treatments for patients admitted to the intensive care unit. We also excluded RCTs that did not report any critical outcome and that reported different analyses in both documents (e.g., interim analysis reported in the preprint and final analysis reported in the journal article).

### Linking preprint and subsequent journal article

The linkage between the preprint and journal article was performed as part of the COVID-NMA living systematic review. The preprint–article linker was developed in collaboration with a research team from the French National Centre for Scientific Research. The tool automatically generated an alert when a preprint was updated or published as a journal article. Pairs of researchers used the tool to identify these subsequent reports and then extracted any additional and/or updated data independently, meeting for consensus to reconcile any disagreements. Consequently, an accurate record of the corresponding preprint and journal publication reports in the COVID-NMA database is available for download as a preprint-publication pair. To identify eligible RCTs, one researcher (MD) retrieved this record from the COVID-NMA database and selected the first preprint posted on a preprint server and the subsequent journal article. When available, we used the online publication date in order to calculate the delay between preprint post and journal article publication. Otherwise, we used the print publication date.

### Data extraction

We retrieved data that were previously extracted in duplicate independently by pairs of researchers, with consensus to resolve disagreements for the COVID-NMA living systematic review: publication type (preprint, journal article), publication date (date that the report was published online, when available), trial registration (prospective, retrospective relative to the start date of the trial), funding type (industry, mixed, public, none, not reported/unclear), study centers (single, multicentric), setting (hospital, outpatient clinic), geographical RCT location according to the World Bank Country Income Classification [15], and intervention details.

For the critical outcome measures under consideration, the number of participants with outcome events and the number of participants analyzed were retrieved. Risk of bias was assessed according to the Cochrane Risk of Bias 2 tool [16] and each outcome result was rated as ‘Low’, or ‘Some concerns’, or ‘High’ risk of bias. Particularly, we considered the overall risk of bias assessments i.e., the highest risk of bias found in any domain for any critical outcome in the trial. The previously extracted data were split into two parts and two researchers (MD, CG) verified these data, meeting for consensus if a discrepancy was found.

### Data synthesis

For the descriptive analysis, frequencies and percentages were calculated for categorical variables, while medians with interquartile ranges (IQRs) were calculated for continuous variables.

We systematically explored whether the number of participants with outcome events, number of participants analyzed, and treatment effect estimates were consistent between preprints and subsequent journal articles for all critical outcomes. The discrepancies between results reported in a preprint and subsequent journal article were classified as (1) change in the estimate of the effect of at least one outcome, (2) change in the direction of the effect, (3) change in statistical significance, and (4) change in the overall conclusion. We also investigated whether the outcomes were deleted or added in the journal articles compared to the preprints. We used R software, [17] with the *metafor* [18] and *forestplot* [19] packages, for all analyses.

## Results

**Fig. 1:**
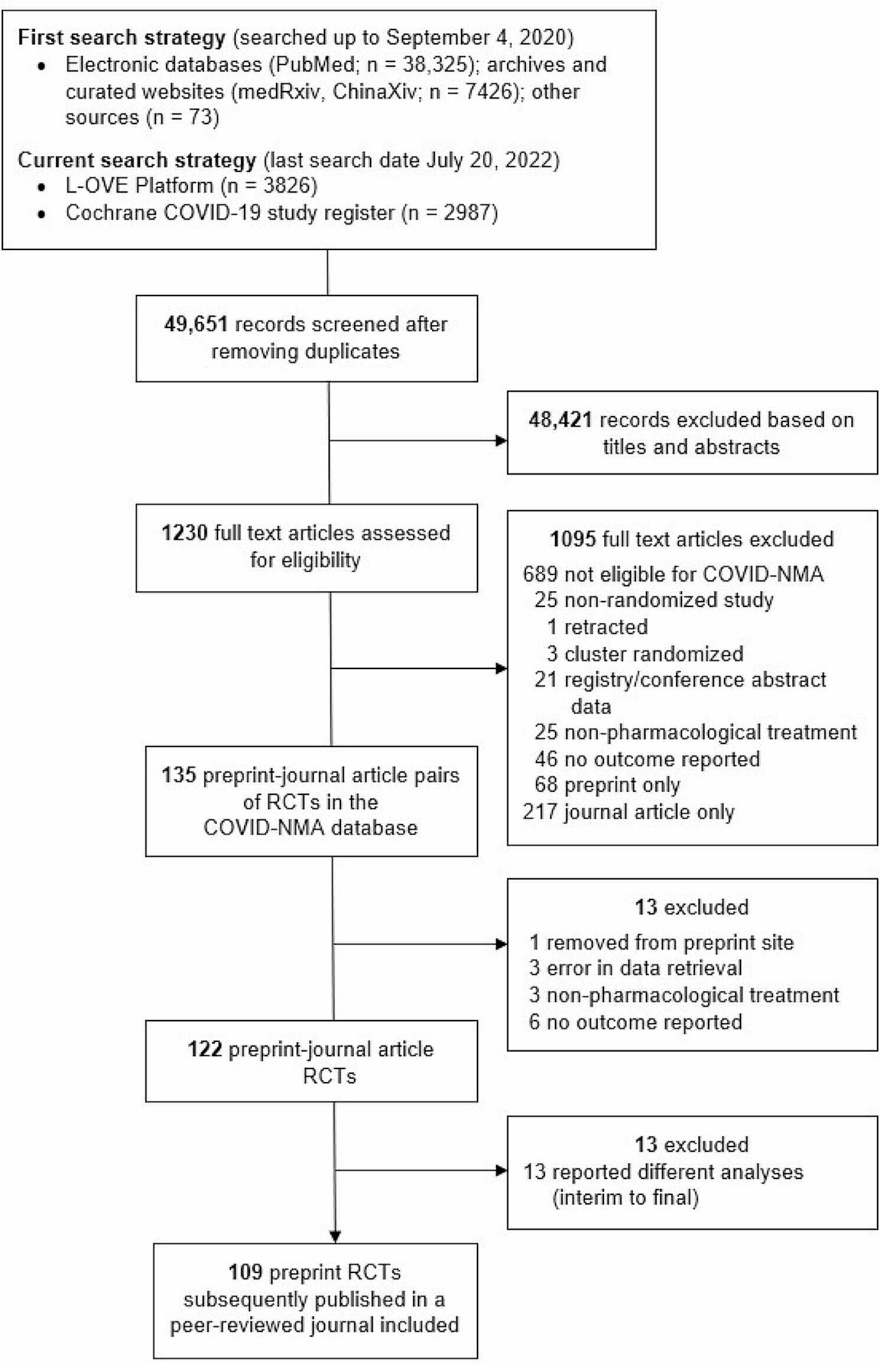
Flowchart of included randomized controlled trials (last search date July 20, 2022)

Of the 49,651 records screened, 1230 were assessed for eligibility and we identified 135 treatment RCTs that were originally posted as a preprint and subsequently published as a journal article. We excluded 26 RCTs because they did not conform to eligibility criteria; one preprint was removed from the preprint server, three RCTs were excluded because there was an error in data retrieval (i.e., they were incorrectly labelled in the COVID-NMA database as a preprint but the data were from trial registry results (n = 2) and from the journal article (n = 1)), three RCTs evaluated non-pharmacological treatments, six RCTs did not report any critical outcomes and 13 RCTs reported interim analysis in the preprint and final analysis in the journal article. Increased sample sizes and longer follow-up and enrolment periods were observed in the final analyses of the subsequent journal articles compared to the interim analyses of the preprints. Overall, 109 RCTs were included in the analysis (Fig. 1).

Characteristics of preprints that were subsequently published in a journal article are presented in Table 1. The median delay between preprint and peer-reviewed journal article was 121 (IQR, 73–187) days. The median sample size was 150 (IQR, 71–464) participants, 76% of RCTs had been prospectively registered, 60% received industry or mixed funding, 72% were multicentric trials. The overall risk of bias assessed was rated as ‘some concern’ for 80% of RCTs.

**Fig. 2:**
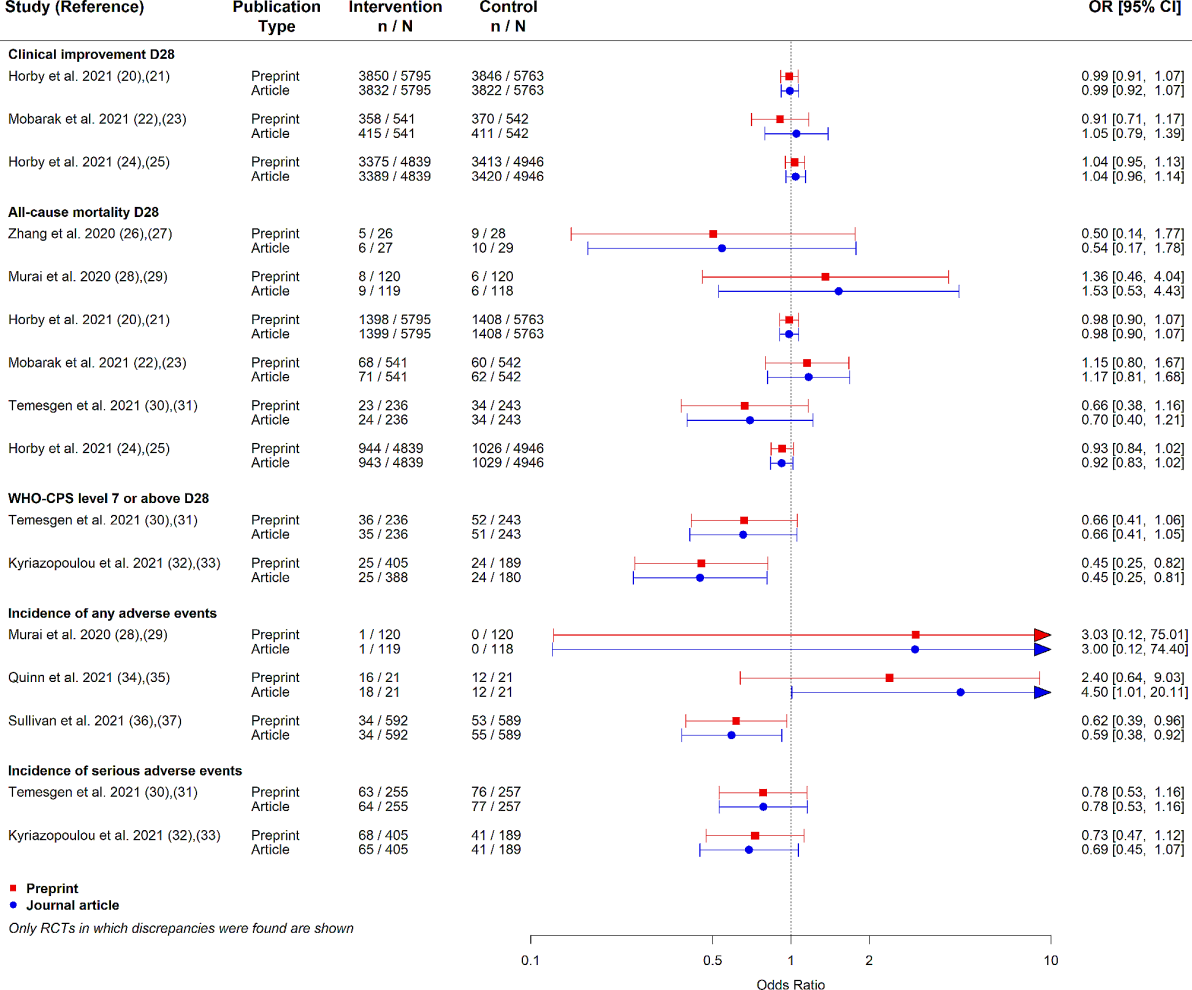
Discrepancy in effect estimates between preprint and subsequent journal article of COVID-19 RCTs. RCT, randomized controlled trial; n, number of participants with outcome events; N, number of participants analyzed; CI, confidence interval; D28, day 28; article, peer-reviewed journal; WHO-CPS, World Health Organization Clinical Progression Score

Of the 109 preprint–article pairs of RCTs, 81 were consistent for all outcomes. We found six RCTs with at least one outcome missing in the journal article, and 14 RCTs with at least one outcome added in the journal article compared to the preprint. There were nine RCTs that had at least one outcome with a change in the number of participants with outcome events or the number of participants analyzed, which yielded a minor change in the estimate of the effect (Fig. 2) [20–37]. There was one RCT with a change in the direction of the effect. No changes in the statistical significance or overall conclusions between preprint and journal article were observed for any RCT.

Characteristics of the preprints that were never published in a peer-reviewed journal are compared to those that were published (Additional file Table S2). Generally, we found that basic characteristics of RCTs initially posted as preprints were similar between those that were subsequently published and those that were not.

**Table 1:** Characteristics of preprint**–**article RCTs

Characteristics		Preprint–Article RCTs n = 109 (%)	RCTs with consistent data n = 81 (%)	RCTs with added/ deleted outcomes n = 20^*^ (%)	RCTs with change in effect estimate n = 9^*^ (%)
Sample size, median (IQR)		150 (71–464)	149 (66–420)	129 (85–663)	606 (240–1225)
Delay^†^, median (IQR)		121 (73–187)	128 (79–187)	91 (27–153)	127 (99–210)
Registration timing, n (%)	Prospective	83 (76)	62 (77)	14 (70)	8 (89)
	Retrospective	25 (23)	18 (22)	6 (30)	1 (11)
	Not reported/unclear	1 (1)	1 (1)	0	0
Funding type, n (%)	Industry/mixed	65 (60)	45 (56)	16 (80)	5 (56)
	Public	34 (31)	28 (35)	1 (5)	4 (44)
	Others	10 (9)	8 (10)	3 (15)	0
Study centers, n (%)	Single	30 (28)	28 (35)	2 (10)	0
	Multicenter	79 (72)	53 (65)	18 (90)	9 (100)
Overall risk of bias^⁑^, n (%)	Low	13 (12)	7 (9)	6 (30)	1 (11)
	Some concerns	87 (80)	66 (81)	13 (65)	8 (89)
	High	9 (8)	8 (10)	1 (5)	0
Setting, n (%)	Hospital	93 (85)	67 (83)	18 (90)	8 (89)
	Outpatient clinic	16 (15)	14 (17)	2 (10)	1 (11)
Geographical location^‡^, n (%)	High-income countries	42 (39)	30 (37)	8 (40)	5 (56)
	Low-/middle-income countries	49 (45)	39 (48)	7 (35)	3 (33)
	Countries of different income levels	18 (17)	12 (15)	5 (25)	1 (11)
Preprint post^§^, n (%)	< 6 months	21 (19)	14 (17)	6 (30)	1 (11)
	6–12 months	45 (41)	32 (40)	10 (50)	3 (33)
	> 12 months	43 (39)	35 (43)	4 (20)	5 (56)

RCT, randomized controlled trial; mixed, industry and public funding; others, no funding/not reported/unclear

^*^ One RCT had an outcome added in the journal article and outcomes with changes in the effect estimate

^†^ Number of days between preprint post and journal article publication online

^⁑^ Highest risk of bias assessed for any outcome in any domain

^‡^ World Bank Country Income Classifications [15]

^§^ Relative to March 2020 i.e., start of the pandemic

## Discussion

In this study, we analyzed the consistency in treatment effect estimates between RCTs first available as a preprint and subsequently published in a peer-reviewed journal. We found only trivial discrepancies between COVID-19 preprints and subsequent journal articles in most pharmacological treatment RCTs. Nevertheless, some outcomes were added and deleted in the journal articles compared with the preprints and one trial showed a change in the direction of effect between preprint and subsequent journal article.

Our study findings demonstrate substantial agreement with the conclusions of other COVID-19 studies. In a retrospective review of 74 RCTs included in a living network meta-analysis [38, 39] up to August 2021, Zeraatkar et al. did not observe important discordance between the first preprint and subsequent journal article [10]. The cross-sectional study by Bero et al. found only marginal changes to outcomes reporting and spin between 67 preprint–article pairs of studies published between March and October 2020 [9]. In contrast, in a meta-research study of preventive, therapeutic, or post-acute care interventions for COVID-19, Oikonomidi et al. found significant changes in results and abstract’s conclusions in 55% of the sample of 66 preprint–article studies published up to August 2020 [7].

While over half (58%) of preprints are subsequently published in a peer-reviewed journal [40], the fact is that some will remain unpublished, due to journal rejection because of poor methodological and statistical quality or, in rare cases, lack of submission. Based on this, some suggest that preprints should be excluded from meta-analyses [41]. Thus, as part of objective two of our protocol, we conducted a meta-epidemiological study, selecting 37 meta-analyses at different timepoints that included both preprint and journal article RCTs [42].

### Strengths and limitations

We assessed the consistency of results between preprint and journal article pairs of RCTs, as significant changes found in the subsequent journal article bring the reliability of preprint data into question. Furthermore, our data were retrieved from a large living systematic review (COVID-NMA). COVID-NMA employed a validated, comprehensive search strategy to identify all relevant evidence.

There are some limitations of our assessment. Firstly, this research was conducted on COVID-19 RCTs, so results may not be generalizable to other fields and study types. In non-COVID-19-related studies, Carneiro et al. [43] determined that preprints were lacking in reporting quality but, on average, the quality of reporting between preprints and subsequent journal articles was comparable. Another study found small differences in journal article conclusions of 7.2% of non-COVID-19–related and 17.2% of COVID-19–related abstracts compared to the preprint [44]. Secondly, for those preprints that were never published in a journal, we could not evaluate whether peer review prevented journal publication due to unsupported conclusions. Nevertheless, we found that trial characteristics were generally similar between preprints that were subsequently published in peer-reviewed journals and those that remained unpublished. Finally, our study is limited to the decisions of the living review. For example, protocol revisions could affect the sample composition.

## Conclusion

We identified changes in effect estimates in 8% of COVID-19 randomized controlled trials between preprint and subsequent journal article. Some outcomes were deleted or added in the journal articles; therefore, it is important to retrieve both documents and explore reasons for discrepancies. Certainly, a critical approach should be adopted when using results from preprints due to the lack of peer review.

### Electronic supplementary material

Below is the link to the electronic supplementary material.

**Supplementary Material 1:** Definitions of trial characteristics; Methods S1; Table S1; Table S2; Figure S1

## Data Availability

The data and code used during the current study are available at https://github.com/MDavids0n/Preprint_Journal.
