# Supplementary material for: Comparison of effect estimates between preprints and peer-reviewed journal articles of COVID-19 trials
Source: BMC Med Res Methodol. 2024 Jan 11;24:9. doi: 10.1186/s12874-023-02136-8 (PMC10782611; doi:10.1186/s12874-023-02136-8)
Supplement: Supplementary file 1 — Supplementary Material 1: Definitions of trial characteristics; Methods S1; Table S1; Table S2; Figure S1 [file 12874_2023_2136_MOESM1_ESM.docx]

**Additional file 1**

Definitions of trial characteristics

**Methods S1.** Search Strategy

**Table S1:** Description of RCTs that were retracted or removed before the search date

**Table S2.** Characteristics of preprint RCTs

**Figure S1.** Relationship between delay to publication and discrepancies in preprint-article RCTs

**References S1**

Definitions of trial characteristics

Registration timing

- Prospective – registered before the start date of the trial
- Retrospective – registered after the start date of the trial

Funding type

- Mixed – received industry and public funding
- Others – received no funding or funding was not reporting or unclearly reported

Geographical location (classified using the World Bank Country Income Classifications^2^)

- Low-/middle-income country – countries classified as:
  - low-income – GNI per capita, calculated using the World Bank Atlas method, of $1,135 or less in 2022
  - lower middle-income – GNI per capita between $1,136 and $4,465

and/or

- - upper middle-income – GNI per capita between $4,466 and $13,845
- High-income country – GNI per capita of $13,846 or more

Risk of bias (classified according to the Cochrane Risk of Bias 2.0 tool^1^)

Using signalling questions, risk of bias is assessed for all outcomes across five domains – 1) Randomization, 2) Deviations from the intervention, 3) Missing outcome data, 4) Measurement of the outcome and 5) Selection of the reported result. An algorithm analyzed the responses to these signalling questions to generate an assessment for each domain, which were categorized as "low," "some concerns," or "high."

- Overall risk of bias – highest risk of bias found in any domain for an outcome in the trial

Methods S1

***Search strategy***

The initial search strategy was developed with Robin Featherstone, Information Specialist, at the Cochrane Editorial & Methods Department and evolved following assessment of bibliographic databases. The search was updated on September 4, 2020 following an evaluation of the sensitivity of the L-OVE platform and Cochrane COVID-19 Study Register by Pierre et al^3^, which identified all RCTs identified through the initial extensive search strategy.

Electronic searches

- The L-OVE platform (<https://app.iloveevidence.com/covid19>), searched every working day since 4 September 2020. Complete data sources and search methods are available at <https://app.iloveevidence.com/covid19/methods>.
- The Cochrane COVID-19 Study Register (<https://covid-19.cochrane.org/>), searched every working day since 4 September 2020. Complete data sources and search methods are available at <https://community.cochrane.org/about-covid-19-study-register>.

Reference sections of included trial reports were not checked for additional articles as the living search process identified COVID-19 trial reports prospectively from the point of trial registration.

The Retraction Watch Database was also searched for retracted studies (<https://retractionwatch.com/retracted-coronavirus-covid-19-papers/>).

Below we describe our initial search strategy and secondary sources.

**First Period of search**

Up to September 2020, we relied on the following sources:

| **PubMed (MEDLINE)** | (2019 nCoV[tiab] OR 2019nCoV[tiab] OR corona virus[tiab] OR corona viruses[tiab] OR coronavirus[tiab] OR coronaviruses[tiab] OR COVID[tiab] OR COVID19[tiab] OR nCov 2019[tiab] OR SARSCoV2[tiab] OR SARS CoV-2[tiab] OR SARSCoV2[tiab] OR SARSCoV-2[tiab] OR "COVID-19"[Mesh] OR "COVID-19 Testing"[Mesh] OR "COVID-19 Vaccines"[Mesh] OR "Coronavirus"[Mesh:NoExp] OR "SARS-CoV-2"[Mesh] OR "COVID-19"[nm] OR "COVID-19 drug treatment"[nm] OR "COVID-19 diagnostic testing"[nm] OR "COVID-19 serotherapy"[nm] OR "COVID-19 vaccine"[nm] OR "LAMP assay"[nm] OR "severe acute respiratory syndrome coronavirus 2"[nm] OR "spike protein, SARSCoV-2"[nm]) NOT ("animals"[mh] NOT "humans"[mh]) NOT (editorial[pt] OR newspaper article[pt]) |
| --- | --- |
| **Embase.com** | ((('coronaviridae'/de OR 'coronavirinae'/de OR 'coronaviridae infection'/de OR 'coronavirus disease 2019'/exp OR 'coronavirus infection'/de OR 'SARS-related coronavirus'/de OR 'Severe acute respiratory syndrome coronavirus 2'/exp OR '2019 nCoV':ti,ab,kw OR 2019nCoV:ti,ab,kw OR ((corona* OR corono*) NEAR/1 (virus* OR viral* OR virinae*)):ti,ab,kw OR coronavir*:ti,ab,kw OR coronovir*:ti,ab,kw OR COVID:ti,ab,kw OR COVID19:ti,ab,kw OR HCoV*:ti,ab,kw OR 'nCov 2019':ti,ab,kw OR 'SARS CoV2':ti,ab,kw OR 'SARS CoV 2':ti,ab,kw OR SARSCoV2:ti,ab,kw OR 'SARSCoV 2':ti,ab,kw) NOT (('animal experiment'/de OR 'animal'/exp) NOT ('human'/exp OR 'human experiment'/de))) NOT 'editorial'/it) NOT ([medline]/lim OR [pubmed-not-medline]/lim) AND [1-12-2019]/sd |
| **Cochrane Central Register of Controlled trials (CENTRAL)** | 1 ("2019 nCoV" OR 2019nCoV OR "corona virus*" OR coronavirus* OR COVID OR COVID19 OR "nCov 2019" OR "SARS-CoV2" OR "SARS CoV-2" OR SARSCoV2 OR "SARSCoV-2"):TI,AB AND CENTRAL:TARGET  2 Coronavirus:MH AND CENTRAL:TARGET  3 Coronavirus:EH AND CENTRAL:TARGET  4 #1 OR #2 OR #3  5 2019 TO 2021:YR AND CENTRAL:TARGET  6 #5 AND #4  7 INSEGMENT  8 #6 NOT #7 |
| **ClinicalTrials.gov** | COVID‐19 OR 2019‐nCoV OR SARS‐CoV‐2 OR coronavirus |
| **WHO ICTRP** | COVID OR 2019‐nCoV OR SARS‐CoV‐2 OR coronavirus OR corona virus |
| **MedRχiv** | A curated list of records for COVID-19 and SARS-CoV-2 is available at https://connect.biorxiv.org/relate/ content/181. Note that this list also includes sources listed in bioRχiv, but we only screened the sources published on MedRχiv. |
| **Chinaχiv** | **Searched** up to 7 April 2020 |

- We also searched The Cochrane Covid-19 Study Register used as quality control and Epistemonikos L·OVE COVID-19 platform from June 2020.

**Second Period (from September 2020)**

Since September 2020, we relied on the following sources:

*1) The* Living OVerview of Evidence (*L-OVE) platform*

Details related to the search performed by this platform and the process is available here https://app.iloveevidence.com/loves/5e6fdb9669c00e4ac072701d?population=5e7fce7e3d05156b5f5e032a&intervention_variable=603b9fe03d05151f35cf13dc&section=methods&classification=all.

In brief, the Living OVerview of Evidence (L·OVE) was built, and is maintained, by systematic searches in multiple databases, trial registries and preprint servers. The following sources are regularly searched:

- Pubmed/medline (updated several times a day)
- EMBASE (updated weekly)
- CINAHL (updated weekly)
- PsycINFO (updated weekly)
- LILACS (Latin American & Caribbean Health Sciences Literature) (updated weekly)
- Wanfang Database (updated every 2 weeks)
- CBM - Chinese Biomedical Literature Database (updated every 2 weeks)
- CNKI - Chinese National Knowledge Infrastructure (updated every 2 weeks)
- VIP - Chinese Scientific Journal Database (updated every 2 weeks)
- IRIS (WHO Institutional Repository for Information Sharing) (updated weekly)
- IRIS PAHO (PAHO Institutional Repository for Information Sharing)) (updated weekly)
- IBECS - Índice Bibliográfico Español en Ciencias de la Salud (Spanish Bibliographic Index on Health Sciences) (updated weekly)
- Microsoft Academic (last searched: 23 August 2021)
- ICTRP Search Portal (updated daily)
- Clinicaltrials.gov (updated daily)
- ISRCTN registry (updated daily)
- Chinese Clinical Trial Registry (updated daily)
- IRCT - Iranian Registry of Clinical Trials (updated daily)
- EU Clinical Trials Register: Clinical trials for covid-19 (updated daily)
- NIPH Clinical Trials Search (Japan) - Japan Primary Registries Network (JPRN) (JapicCTI, JMACCT CTR, jRCT, UMIN CTR) (updated daily, via ICTRP search portal)
- UMIN-CTR - UMIN Clinical Trials Registry (updated daily, via ICTRP search portal)
- JRCT - Japan Registry of Clinical Trials (updated daily, via ICTRP search portal)
- JAPIC Clinical Trials Information (updated daily, via ICTRP search portal)
- Clinical Research Information Service (CRiS), Republic of Korea (updated daily, via ICTRP search portal)
- ANZCTR - Australian New Zealand Clinical Trials Registry (updated daily, via ICTRP search portal)
- ReBec - Brazilian Clinical Trials Registry (updated daily, via ICTRP search portal)
- CTRI - Clinical Trials Registry - India (updated daily, via ICTRP search portal)
- RPCEC - Cuban Public Registry of Clinical Trials (updated daily, via ICTRP search portal)
- DRKS - German Clinical Trials Register (updated daily, via ICTRP search portal)
- LBCTR - Lebanese Clinical Trials Registry (updated daily, via ICTRP search portal)
- TCTR - Thai Clinical Trials Registry (updated daily, via ICTRP search portal)
- NTR - The Netherlands National Trial Register (updated daily, via ICTRP search portal)
- PACTR - Pan African Clinical Trial Registry (updated daily, via ICTRP search portal)
- REPEC - Peruvian Clinical Trial Registry (updated daily, via ICTRP search portal)
- SLCTR - Sri Lanka Clinical Trials Registry (updated daily, via ICTRP search portal)
- medRxiv (updated several times a day)
- bioRxiv (updated several times a day)
- SSRN Preprints (updated several times a day)
- ChinaXiv (updated every 2 weeks)
- SciELO Preprints (updated weekly)
- Research Square (updated daily)

*2) The Cochrane Covid-19 Study Register*

Details related to the search performed by this register and the process are described here: https://community.cochrane.org/about-covid-19-study-register. It is a specialised register built within the Cochrane Register of Studies (CRS) and is maintained by Cochrane Information Specialists. The register contains study reports from several sources, including:

- daily searches of PubMed
- daily searches of ClinicalTrials.gov
- weekly searches of Embase.com
- weekly searches of the WHO International Clinical Trials Registry Platform (ICTRP)
- weekly searches of medRxiv
- monthly searches of the Cochrane Central Register of Controlled Trials (CENTRAL)

*3) Retraction Watch*

We also searched the Retraction Watch Database for retracted studies ([retractionwatch.com/retracted-coronavirus-covid-19-papers/](file:///C:\Users\kapp\Desktop\Final_BMC\Re-submission\Adapted\Final_IB\retractionwatch.com\retracted-coronavirus-covid-19-papers\)).

***Screening***

We used an Excel spreadsheet to document search dates and citations identified. The Rayyan QCRI software (https://www.rayyan.ai/) was used to manage the records and data obtained for screening. Duplicates were removed, then title/abstract screening and full-text consideration were done by pairs of researchers, in duplicate and independently, with a third researcher resolving any disagreements.

**Table S1:** Description of RCTs that were retracted or removed before the search date

| **Reference** | **Treatment** | **Publication type** | **Registration number** | **Retracted date** | **Reason for retraction** | **Link to retraction note** |
| --- | --- | --- | --- | --- | --- | --- |
| Bosaeed M, SSRN, 2021 | Favipiravir | Preprint | NCT04392973 | Not reported | Not reported | https://papers.ssrn.com/sol3/papers.cfm?abstract_id=3829663 |
| Dabbous HM, Arch Virol, 2021 | Favipiravir | Journal article | NCT04351295 | 2021-11-22 | Methodological concerns | https://www.ncbi.nlm.nih.gov/pmc/articles/PMC8608235/ |
| Dabbous HM, Sci Rep, 2021 | Favipiravir | Preprint to Journal article | NCT04349241 | 2021-09-18 | Methodological concerns | https://www.ncbi.nlm.nih.gov/pmc/articles/PMC8462367/ |
| Elgazzar A, Research Square, 2021 | Ivermectin | Preprint | NCT04668469 | 2021-07-14 | Potential fabrication and plagiarism | https://grftr.news/why-was-a-major-study-on-ivermectin-for-covid-19-just-retracted/ |
| Ghati N, SSRN, 2021 | Atorvastatin and Aspirin | Preprint | CTRI/2020/07/026791 | Not reported | Not reported | https://papers.ssrn.com/sol3/papers.cfm?abstract_id=3820512 |
| McCoy J, Frontiers, 2021 | Proxalutamide | Preprint to Journal article | NCT04446429 | 2022-06-08 | Methodological concerns | https://www.ncbi.nlm.nih.gov/pmc/articles/PMC9226906/ |
| Pott-Junior H, Toxicology Reports, 2021 | Ivermectin | Journal article | NCT04431466 | 2022-05-02 | Methodological concerns and insufficient reporting | https://www.ncbi.nlm.nih.gov/pmc/articles/PMC9060540/ |
| Samaha AA, Viruses, 2021 | Ivermectin | Journal article | Not reported | 2021-09-18 | Error and potential falsification | https://www.ncbi.nlm.nih.gov/pmc/articles/PMC8577689/ |
| Youssef J, SSRN, 2021 | Aviptadil | Preprint | NCT04311697 | Not reported | Not reported | https://papers.ssrn.com/sol3/papers.cfm?abstract_id=3794262 |

**Table S2:** Characteristics of unpublished and published RCTs

| **Characteristics** | | **Total preprint RCTs**  n = 177 (%) | **Preprint–Article RCTs**  n = 109 (%) | **Preprint only RCTs**  n = 68 (%) |
| --- | --- | --- | --- | --- |
| Sample size, median (IQR) | | 120 (60–388) | 150 (71–464) | 85 (49–287) |
| Registration timing, n (%) | Prospective | 133 (75) | 83 (76) | 50 (74) |
|  | Retrospective | 42 (24) | 25 (23) | 17 (25) |
|  | Not reported/unclear | 2 (1) | 1 (1) | 1 (1) |
| Funding type,  n (%) | Industry/mixed | 101 (57) | 65 (60) | 36 (53) |
|  | Public | 57 (32) | 34 (31) | 23 (34) |
|  | Others | 19 (11) | 10 (9) | 9 (13) |
| Study centers, n (%) | Single | 56 (32) | 30 (28) | 26 (38) |
|  | Multicenter | 121 (68) | 79 (72) | 42 (62) |
| Overall risk of bias^⁑^, n (%) | Low | 18 (10) | 13 (12) | 5 (7) |
|  | Some concerns | 141 (80) | 87 (80) | 54 (79) |
|  | High | 18 (10) | 9 (8) | 9 (13) |
| Setting, n (%) | Hospital | 142 (80) | 93 (85) | 49 (72) |
|  | Outpatient clinic | 35 (20) | 16 (15) | 19 (28) |
| Geographical location^‡^,  n (%) | High-income countries | 73 (41) | 42 (39) | 31 (46) |
|  | Low-/middle-income countries | 79 (45) | 49 (45) | 30 (44) |
|  | Countries of different income levels | 25 (14) | 18 (17) | 7 (10) |
| Preprint post^§^, n (%) | < 6 months | 29 (16) | 21 (19) | 8 (12) |
|  | 6–12 months | 57 (32) | 45 (41) | 12 (18) |
|  | > 12 months | 91 (51) | 43 (39) | 48 (71) |

*RCT, randomized controlled trial; Preprint only, preprint RCTs that were never published; Mixed, industry and public funding; Others, no funding/not reported/unclear*

*^†^Number of days between preprint post and journal article publication online*

*^⁑^Highest risk of bias assessed for any outcome in any domain*

*^‡^World Bank Country Income Classifications* ^2^

*^§^Relative to March 2020 i.e., start of the pandemic*

**
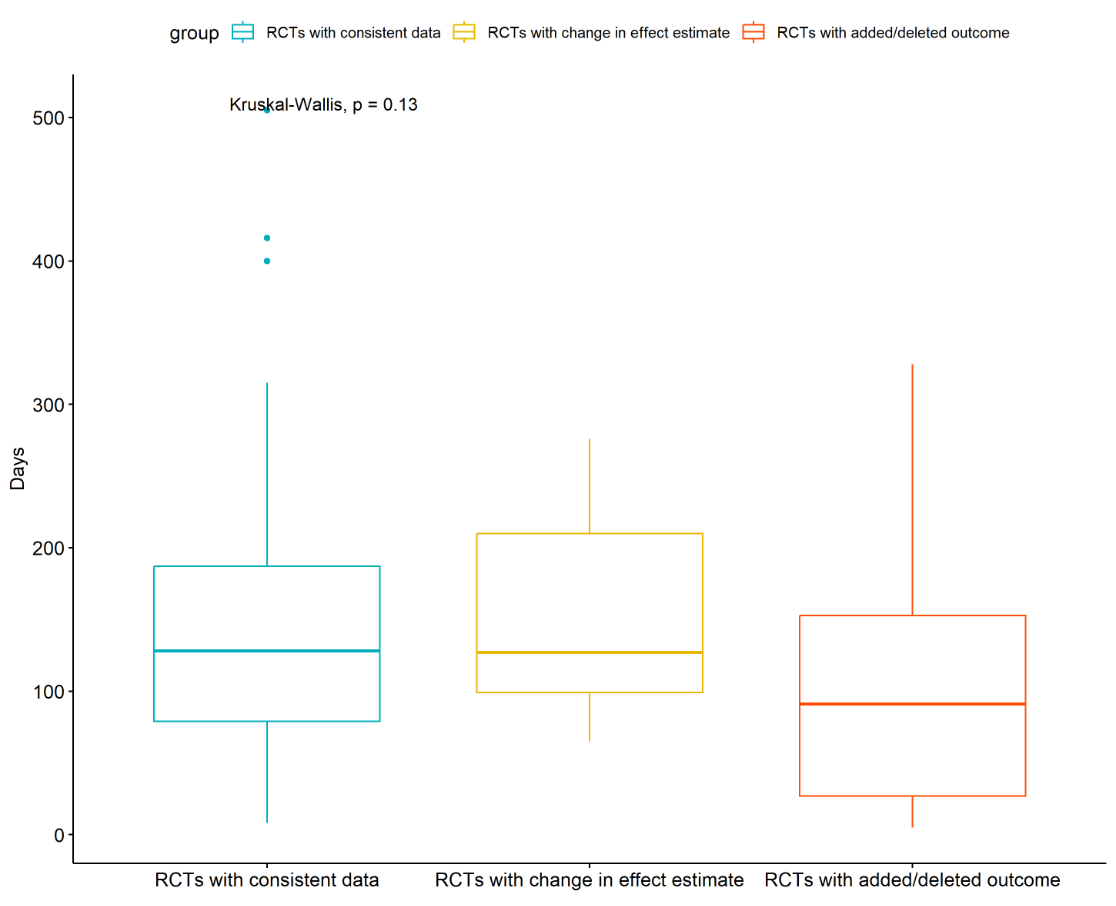
**

**Figure S1:** Relationship between delay to publication and discrepancies in preprint-article RCTs

References S1

1. Sterne JAC, Savović J, Page MJ, et al. RoB 2: a revised tool for assessing risk of bias in randomised trials. *BMJ*. 2019;366:l4898. doi:10.1136/bmj.l4898

2. World Bank Country and Lending Groups – World Bank Data Help Desk. Accessed November 17, 2022. https://datahelpdesk.worldbank.org/knowledgebase/articles/906519-world-bank-country-and-lending-groups

3. Pierre O, Riveros C, Charpy S, Boutron I. Secondary electronic sources demonstrated very good sensitivity for identifying studies evaluating interventions for COVID-19. *J Clin Epidemiol*. 2022;141:46-53. doi:10.1016/j.jclinepi.2021.09.022
